# Schiff-Base-Engineered Fibrous Mesoporous Silica (KCC-1) as an Efficient Sorbent for Dispersive Solid-Phase Extraction of Trace Ni(II) and Cd(II) from Water

**DOI:** 10.3390/nano16150903

**Published:** 2026-07-23

**Authors:** Yassin T. H. Mehdar, Awadh O. Alsuhaimi, Sultan K. Alharbi, Manal A. Almalki, Khaled M. AlMohaimadi, Bandar R. Alsehli, Khalid Althumayri, Bader M. Altayeb, Belal H. M. Hussein

**Affiliations:** 1 Chemistry Department, Faculty of Science, Taibah University, Medina Munwarah 42353, Saudi Arabiasbdrani@taibahu.edu.sa (S.K.A.); btayeb@taibahu.edu.sa (B.M.A.); belalnegm@science.suez.edu.eg (B.H.M.H.); 2 Ministry of Education, Medinah Al-Munwwarah 42314, Saudi Arabia; 3 Department of Chemistry, Faculty of Science, Suez Canal University, Ismailia 41522, Egypt

**Keywords:** fibrous mesoporous silica nanoparticles, KCC-1, O-vanillin, adsorption kinetics and isotherms, dispersive solid phase extraction

## Abstract

The development of reusable nanomaterials for the extraction of trace-metals from complex matrices remains challenging because strong metal-chelating functionalities often hinder desorption and regeneration, whereas weaker binding sites compromise selectivity and enrichment efficiency. This limitation has been addressed by designing a ligand-engineered fibrous mesoporous silica nanomaterial (Van-KCC-1) via the integration of the unique structural features of KCC-1 with an o-vanillin-derived Schiff-base chelator. The material was synthesized throughout the chemical grafting of 3-aminopropyltriethoxysilane (APTES) onto fibrous mesoporous silica KCC-1, followed by condensation with 3-methoxy-2-hydroxybenzaldehyde (o-vanillin). The successfulness of functionalization and Schiff-base formation were confirmed by X-ray diffraction (XRD), Fourier-transform infrared spectroscopy (FT-IR), Thermogravimetric analysis (TGA), and X-ray photoelectron spectroscopy (XPS). The radially oriented fibrous channels of KCC-1 provide a highly accessible surface that remains available for interaction with the targeted ions even after chemical modification. This architecture facilitates rapid mass transfer and efficient utilization of binding sites, while the incorporated Schiff-base ligand introduces imine, phenolic, and methoxy donor groups capable of selectively and reversibly coordinating Ni(II) and Cd(II). The resulting balance between adsorption strength and desorption efficiency enables both effective metal capture and sorbent reusability. More importantly, the study demonstrates how KCC-1 can serve as a versatile nanosilica scaffold for the incorporation of tailored chelating ligands without sacrificing structural accessibility. The functionalized nanomaterial was evaluated as a dispersive solid-phase extraction (DSPE) sorbent coupled with inductively coupled plasma optical emission spectrometry (ICP-OES). Under optimized conditions, linear ranges of 0.035–50 μg L^−1^ for Ni(II) and 0.058–50 μg L^−1^ for Cd(II) were obtained, with limits of detection of 0.011 and 0.019 μg L^−1^, respectively. The method exhibited excellent precision (relative standard deviation ≤ 3.6%) and recoveries of 92.00–98.83% in certified reference materaisl (NIST CRM 1643d), mineral water, tap water and synthetic wastewater. In addition, the nanochelator has retained more than 87% of its initial sorption efficiency after six adsorption–desorption cycles and showed minimal interference from common coexisting ions. These findings establish Van-KCC-1 as an efficient, selective, and reusable DSPE sorbent in the determination of trace-metals while highlighting the broader potential of fibrous mesoporous silica KCC-1 as a platform for the rational design of next-generation chelated nanomaterials.

## 1. Introduction

The contamination of aquatic systems with heavy metals is a persistent environmental and public-health problem because metal ions do not biodegrade, accumulate in living organisms, and enter drinking-water sources through mining, electroplating, battery production, pigment manufacture, metal finishing, and untreated industrial effluents [1,2]. Cadmium and nickel are especially critical analytical targets: Cd(II) is firmly associated with kidney damage, skeletal disorders, and carcinogenesis, whereas Ni(II) is released from stainless-steel processing, electroplating, catalyst production, and battery-related activities and causes toxic and allergenic effects at elevated exposures [3,4,5,6,7]. Both metals persist at concentrations well below the sensitivity limits of direct instrumental measurement in typical environmental waters, making trace-level determination a practical challenge for water-quality surveillance.

Direct instrumental determination of Cd(II) and Ni(II) in environmental waters is routinely limited by low analyte concentrations, high dissolved-salt loads, and matrix components that suppress or distort analytical signals. Sample-preparation steps that combine preconcentration with matrix elimination are therefore essential before instrumental detection. Among modern sample-preparation modes, dispersive solid-phase extraction (DSPE) offers practical advantages because the sorbent is dispersed directly in the sample solution, which improves solid–liquid contact, reduces mass-transfer resistance, and shortens extraction time compared with conventional packed-bed solid-phase extraction [8,9,10]. The analytical outcome of DSPE depends primarily on sorbent architecture and surface chemistry: an effective sorbent must adsorb trace analytes quickly from a large aqueous volume and release them quantitatively into a small eluent volume compatible with the detection system.

Ordered mesoporous silicas, such as MCM-41, MCM-48, and SBA-15, and dendritic fibrous mesoporous silica particularly, KCC-1 have been widely investigated as SPE sorbents because their high surface areas, accessible pore networks, and the abundant silanol groups for post-synthetic functionalization [11,12,13,14]. KCC-1 is particularly attractive because its flower-like fibrous architecture provides open radial channels, short diffusion pathways, and high external surface accessibility, which facilitate rapid mass transfer during dispersive extraction. Conventionally ordered silicas such as MCM-41 and SBA-15 possess regular cylindrical channels, and MCM-48 features a three-dimensional cubic pore network. These materials can deliver high adsorption capacities; however, analyte diffusion into cylindrical channels is sometimes restricted by pore blockage after dense organic grafting or slow intraparticle transport, which can limit extraction kinetics in short-contact DSPE protocols. KCC-1 differs by possessing radially oriented fibrous channels and an open flower-like morphology. This architecture exposes binding sites directly to the bulk solution, shortens diffusion pathways, and is therefore inherently better suited to vortex-assisted DSPE, where contact times are typically 20–30 min [11,15,16,17,18,19]. Recent reviews confirm that adsorption selectivity on mesoporous silica improves with tailored organic ligands, but excessive binding strength impairs desorption and limits analytical reuse [20,21].

Several functionalized KCC-1 materials have been reported for trace-metal extraction and preconcentration. Behbahani and Rabiee [22] prepared amine-functionalized KCC-1 and applied it in ultrasonic-assisted dispersive micro-solid-phase extraction of Pb(II) and Cd(II) prior to GFAAS determination. In our previous work [23], KCC-1 was modified with a Schiff-base ligand derived from 3,5-di-tert-butylsalicylaldehyde and successfully employed as a DSPE sorbent for the determination of Pb(II) and Co(II) by ICP-OES. Yue et al. [24] utilized thiol- and amine-functionalized KCC-1-coated stir bars for the simultaneous extraction of several trace metals from seafood samples prior to ICP-MS analysis, whereas Marjani and Mohammadi [16] reported a tetrasulfide-functionalized KCC-1 material with high adsorption capacity toward Hg(II). Collectively, these studies demonstrate that the KCC-1 framework can accommodate a wide range of chelating functionalities while maintaining the rapid mass-transfer characteristics required for analytical sample preparation.

Schiff-base ligands derived from o-vanillin are particularly attractive for metal-ion extraction because they provide a combination of imine nitrogen, phenolic oxygen, and methoxy donor sites capable of coordinating transition-metal ions. Culita et al. [25] demonstrated that o-vanillin-functionalized mesoporous silica exhibited high adsorption capacity toward Pb(II), while Tokay and Bağdat [26] showed that structurally related methoxy-phenol Schiff-base ligands immobilized on silica enabled efficient solid-phase extraction of Cd(II) and Ni(II) with excellent recoveries and low detection limits. These findings highlight the potential of o-vanillin-derived ligands for selective metal-ion recognition. However, the integration of such ligands with the dendritic fibrous KCC-1 architecture for DSPE-based preconcentration of Cd(II) and Ni(II) has not been explored.

In the present work, a dendritic fibrous silica nanochelator (Van-KCC-1) was developed by grafting o-vanillin onto APTES-functionalized KCC-1. The design was guided by the principle that an effective analytical sorbent should provide selective yet reversible metal binding, thereby enabling both efficient extraction and quantitative desorption. The KCC-1 support was selected for its open fibrous structure and favorable mass-transfer characteristics, whereas o-vanillin was chosen to introduce imine nitrogen, phenolic oxygen, and methoxy donor sites for Cd(II) and Ni(II) coordination. The stability of the Schiff-base functionality was also considered, as the ortho-hydroxy aryl salicylaldimine structure formed by o-vanillin is stabilized by intramolecular hydrogen bonding and chelate resonance, improving resistance to hydrolysis during acidic elution. Accordingly, the aim of this study was to synthesize and characterize Van-KCC-1 and evaluate its performance as a DSPE sorbent for the preconcentration of trace Cd(II) and Ni(II) prior to ICP-OES determination in water samples.

## 2. Experimental

### 2.1. Materials and Reagents

All reagents were of analytical grade and used without further purification. Ultrapure water (resistivity ≥ 18.2 MΩ cm) was obtained from a Milli-Q system (Millipore, Billerica, MA, USA) and used throughout. Tetraethyl orthosilicate (TEOS, ≥98%), 3-aminopropyltriethoxysilane (APTES, ≥98%), cetyltrimethylammonium bromide (CTAB, ≥99%), urea, cyclohexane, n-amyl alcohol, and 3-methoxy-2-hydroxybenzaldehyde (o-vanillin, ≥99%) were purchased from Sigma-Aldrich (St. Louis, MO, USA). Toluene and ethanol were obtained from Merck KGaA (Darmstadt, Germany). Hydrochloric acid (HCl, 37%), nitric acid (HNO_3_, 65%), sodium hydroxide (NaOH), and ammonium hydroxide (NH_4_OH, 25%) were supplied by Merck KGaA (Darmstadt, Germany) or Shanghai Chemical Reagent Co. (Shanghai, China). Certified 1000 mg L^−1^ single-element stock solutions of Ni(II) and Cd(II) were obtained from Thermo Fisher Scientific (Waltham, MA, USA) and used for calibration and spiking experiments. Working solutions were freshly prepared by serial dilution with ultrapure water and acidified, when needed, with 1% (*v*/*v*) HNO_3_. All glassware and polypropylene tubes were soaked overnight in 10% (*v*/*v*) HNO_3_ and rinsed thoroughly with ultrapure water before use.

### 2.2. Instrumentation

The synthesized materials were subjected to a comprehensive characterization sequence comprising X-ray diffraction (XRD), surface area and porosity analysis, electron microscopy, Fourier-transform infrared spectroscopy (FT-IR), thermogravimetric analysis (TGA), and X-ray photoelectron spectroscopy (XPS). Wide- and low-angle XRD patterns were recorded to verify the amorphous silica framework and mesoscopic order. Textural properties were measured by N_2_ adsorption–desorption at 77 K using a Micromeritics ASAP 2020 analyzer (Micromeritics Instrument Corp., Norcross, GA, USA). Specific surface area was calculated by the Brunauer–Emmett–Teller (BET) method and pore-size distribution by the Barrett–Joyner–Halenda (BJH) method. Morphology and particle size were examined by field-emission scanning electron microscopy (FE-SEM, Zeiss EM10C, Carl Zeiss AG, Oberkochen, Germany) and transmission electron microscopy (TEM, Tecnai G2 20 S-TWIN, FEI Company, Hillsboro, OR, USA). Functional groups were identified by FT-IR (Thermo Fisher Scientific, Waltham, MA, USA). Thermal stability and grafted organic content were evaluated by TGA (Hitachi NEXTA STA200, Hitachi High-Technologies Corp., Tokyo, Japan). Surface chemical composition was analyzed by XPS (Escalab 250Xi, Thermo Fisher Scientific, Waltham, MA, USA). Metal concentrations were quantified using inductively coupled plasma optical emission spectrometry (ICP-OES, Agilent 5900, Agilent Technologies, Santa Clara, CA, USA). Each adsorption, recovery, and interference experiment was performed in triplicate unless otherwise stated, and results are reported as mean ± standard deviation (SD).

### 2.3. Synthesis of KCC-1

Fibrous mesoporous silica (KCC-1) was synthesized via a reflux-assisted sol–gel method. Briefly, CTAB (0.5 g) and urea (0.5 g) were dissolved in deionized water (40 mL) under magnetic stirring. A solution of TEOS (3 mL) in cyclohexane (30 mL) was added, followed by dropwise addition of n-amyl alcohol (9 mL). The reaction mixture was refluxed under continuous stirring for 48 h. After cooling to room temperature, the resulting solid was collected by centrifugation, washed several times with water and ethanol, and dried at 90 °C overnight. The dried product was calcined at 550 °C for 6 h in air to remove the surfactant template, yielding fibrous KCC-1. The reflux was maintained at 80 °C throughout the 48 h reaction. The fibrous mesoporous KCC-1 was prepared following the microemulsion reflux route reported by Polshettiwar et al. and Bayal et al. [11,15].

### 2.4. Preparation of Schiff-Base Functionalized KCC-1 (Van-KCC-1)

Surface functionalisation was accomplished through a two-step procedure comprising primary amine grafting onto the silica surface, followed by Schiff-base formation with an aldehyde derivative.

(i). Amine Functionalization (H_2_N-KCC-1)

The calcined KCC-1 (1.0 g) was dispersed in dry toluene (50 mL) using sonication. APTES (1.0 mL) was added, and the mixture was refluxed under a nitrogen atmosphere for 24 h. The resulting amine-functionalized material (H_2_N-KCC-1) was collected by centrifugation, washed thoroughly with toluene and ethanol to remove unreacted silane, and dried at 70 °C for 24 h.

(ii). Schiff-Base Modification (Van-KCC-1)

A portion of dry H_2_N-KCC-1 (1.0 g) was redispersed in ethanol (50 mL), followed by the addition of o-vanillin (0.25 g) and a few drops of glacial acetic acid. The mixture was refluxed under stirring for 24 h to allow condensation between surface amine groups and aldehyde functionality, forming imine (–C=N–) linkages.

The resulting Schiff-base-functionalized material (Van-KCC-1) was collected by centrifugation, washed repeatedly with ethanol and water, and dried at 60 °C for 24 h.

### 2.5. Adsorption Experiments

The interaction of Ni(II) and Cd(II) with Van-KCC-1 was evaluated using batch adsorption experiments. Typically, 10 mg of sorbent was dispersed in 100 mL of metal-ion solution (10–250 mg L^−1^) in polyethylene bottles. The pH was adjusted with 0.1 M HCl or 0.1 M NH4OH. The suspensions were agitated at 200 rpm and 25 ± 0.5 °C. At predetermined times, aliquots were withdrawn, centrifuged, filtered when required, acidified with 1% (*v*/*v*) HNO_3_, and analyzed by ICP-OES. Adsorption capacity (qe, mg g^−1^) and removal efficiency (%) were calculated from mass-balance equations using the initial and equilibrium concentrations.

### 2.6. DSPE Procedure

A general DSPE procedure was used for Ni(II) and Cd(II) preconcentration from aqueous samples. The sample pH was adjusted to the optimized value, followed by addition of Van-KCC-1 and vortex-assisted extraction. The sorbent was then separated by centrifugation, and the retained metal ions were desorbed using a small volume of HNO_3_ under vortexing. The eluate was analyzed by ICP-OES, and the preconcentration factor was calculated from the ratio of sample volume to eluent volume. Calibration standards and blanks were processed using the DSPE procedure, while accuracy and precision were evaluated using certified reference material, matrix spikes, and replicate measurements.

## 3. Results and Discussion

### 3.1. Synthesis and Characterization of Van-KCC-1

Fibrous mesoporous silica KCC-1 was prepared using a reflux-assisted sol–gel route and then modified through sequential APTES amination and o-vanillin condensation. The revised characterization discussion follows the requested sequence: XRD, BET surface area, SEM/TEM, FT-IR, TGA, and XPS. This order separates framework preservation, textural change, morphology, surface functional groups, organic loading, and elemental surface composition.

**Figure 1:**
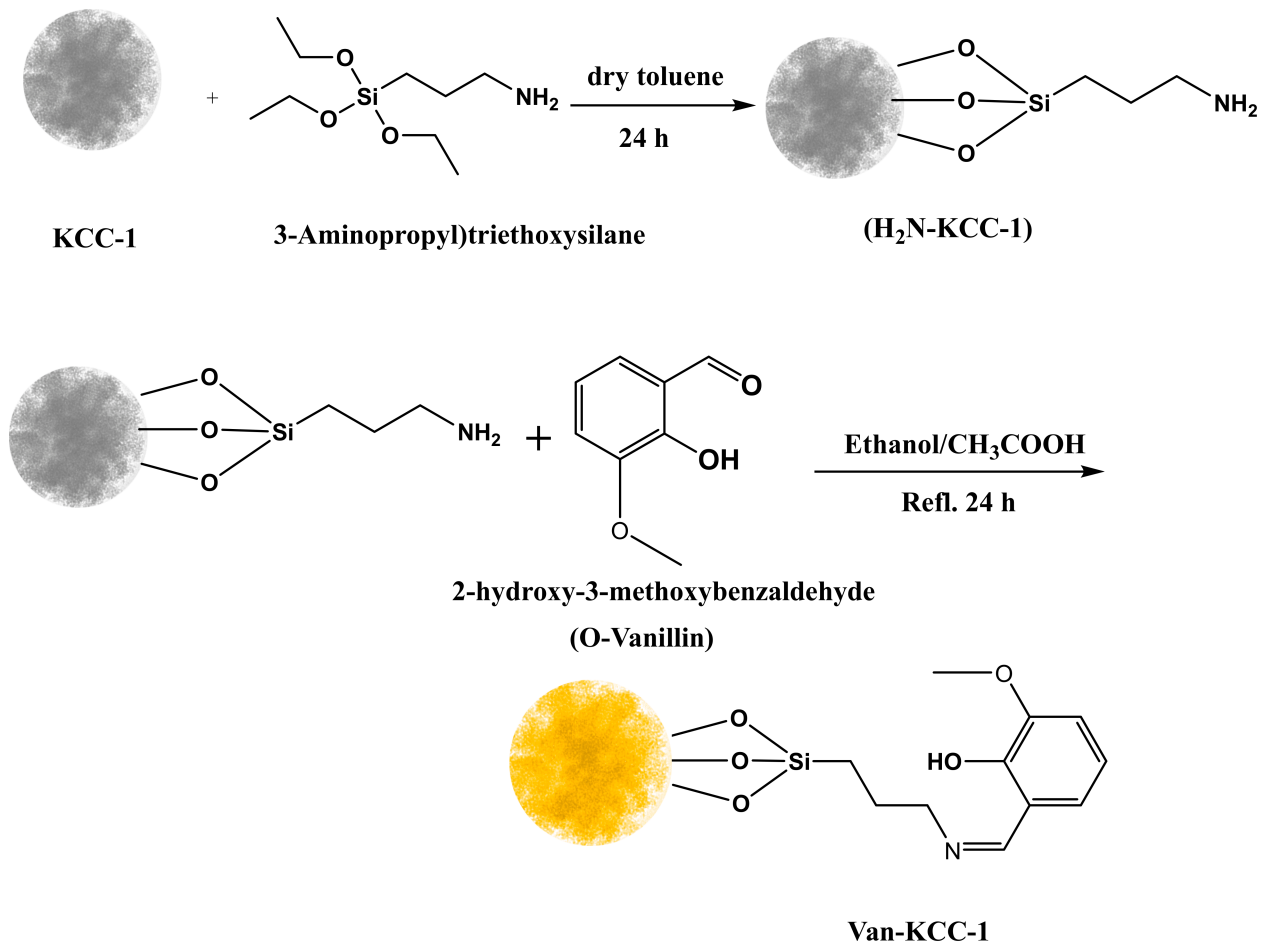
Schematic representation of the preparation of Van-KCC-1. Colour coding distinguishes the three stages of the synthetic sequence: the pristine dendritic fibrous silica framework (white), amine-modified intermediate (white), and Schiff-base functionalized final product (orangey-yellow).

The synthetic pathway is summarized in Figure 1. In the first step, calcined KCC-1 was reacted with APTES under anhydrous conditions to obtain H2N-KCC-1. In the second step, the primary amine groups reacted with o-vanillin to form imine (–C=N–) bonds and the final Schiff-base-functionalized sorbent Van-KCC-1. The selected ligand provides imine nitrogen, phenolic oxygen, and methoxy-substituted aromatic sites that favor moderate and reversible coordination with Ni(II) and Cd(II). This design was selected to avoid the poor elution often observed with very strong chelating surfaces while maintaining sufficient selectivity for trace extraction.

**Figure 2:**
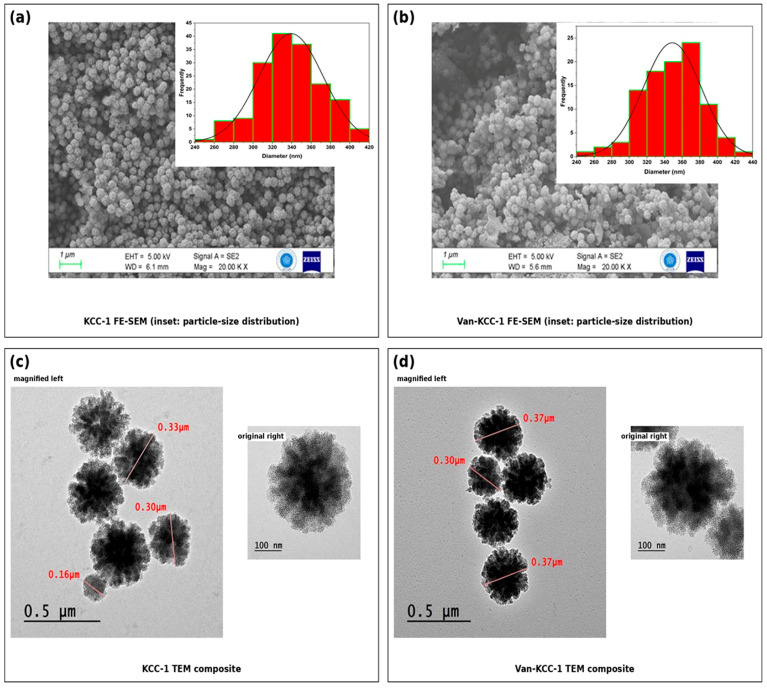
Morphological characterization of KCC-1 and Van-KCC-1: (**a**,**b**) FE-SEM images, particle-size distribution histograms (in the insets), and (**c**,**d**) TEM images showing the flower-like fibrous three-dimensional architecture before and after Schiff-base functionalization.

FE-SEM and TEM images (Figure 2) confirmed that KCC-1 and Van-KCC-1 retained the expected flower-like three-dimensional fibrous morphology. The particle-size distributions shown in the inset in Figure 2a and Figure 2b correspond to KCC-1 and Van-KCC-1, respectively; these panels are therefore identified as particle-size distribution histograms rather than SEM/TEM micrographs. Particle diameters remained mainly within 300–375 nm after functionalization, indicating that post-grafting did not cause severe aggregation or collapse of the dendritic architecture. The TEM images in Figure 2c and Figure 2d confirm the internal radial fibrous channels of KCC-1 and Van-KCC-1, respectively.

**Figure 3:**
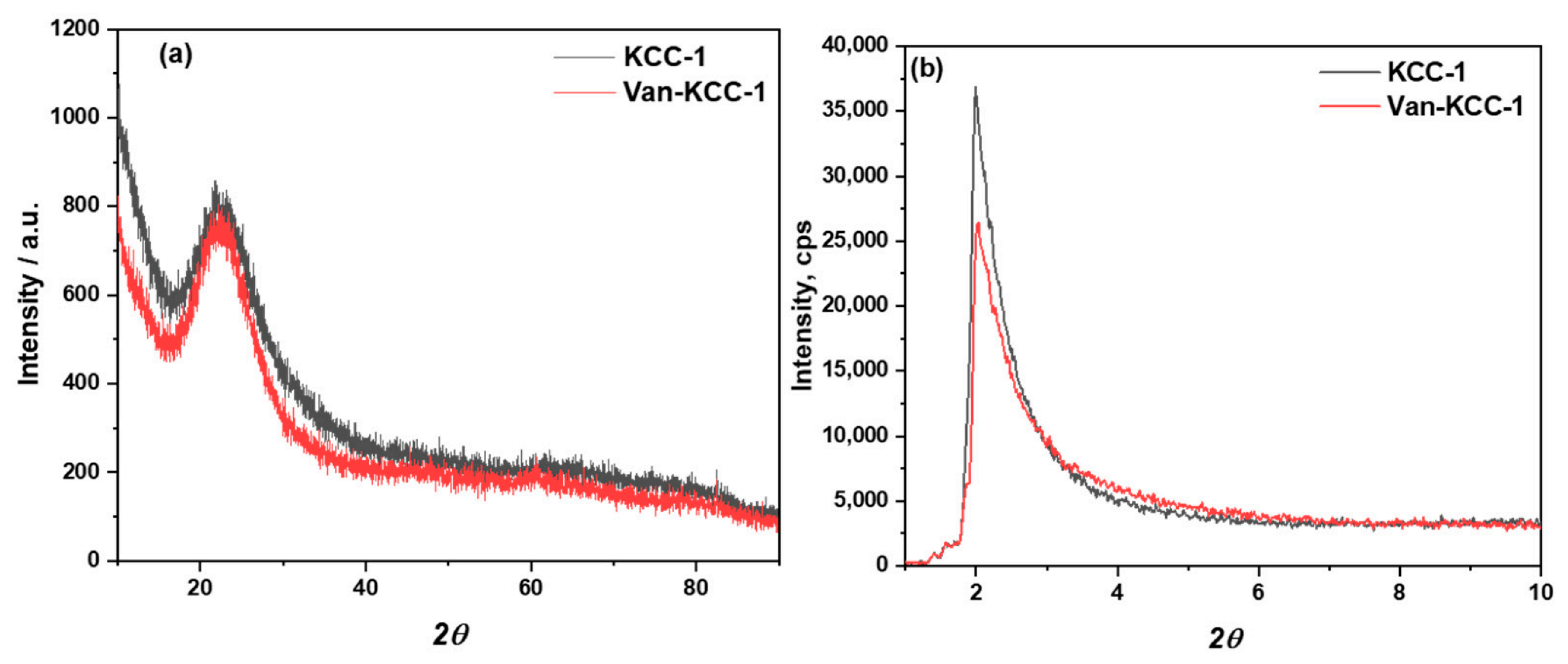
Wide-angle (**a**) and low-angle (**b**) XRD patterns of KCC-1 and Van-KCC-1.

XRD analysis was used first to verify preservation of the silica framework. Wide-angle XRD patterns (Figure 3a) displayed the broad amorphous silica at 2theta ≈ 23° for both KCC-1 and Van-KCC-1, with no crystalline impurity peaks. Low-angle XRD retained the mesoscopic scattering feature of fibrous silica after functionalization, confirming that the post-grafting process modified the surface without destroying the mesostructured framework. The low-angle XRD pattern was recorded from 0.5 to 10 degrees in 2-theta (Figure 3b) and showed the broad low-angle reflection characteristic of the disordered, wormhole-like fibrous mesostructure of KCC-1. This reflection was retained after o-vanillin grafting, which provides direct evidence for the mesoporous structure of both materials. This confirms that the post-grafting process successfully modified the surface without disrupting the mesostructured framework. As shown in Figure 3b, diffraction peaks appear at 2θ = 1.9° and 2.035°, corresponding to interlayer spacings of 4.4 nm and 4.3 nm, respectively (calculated using Bragg’s equation).

**Figure 4:**
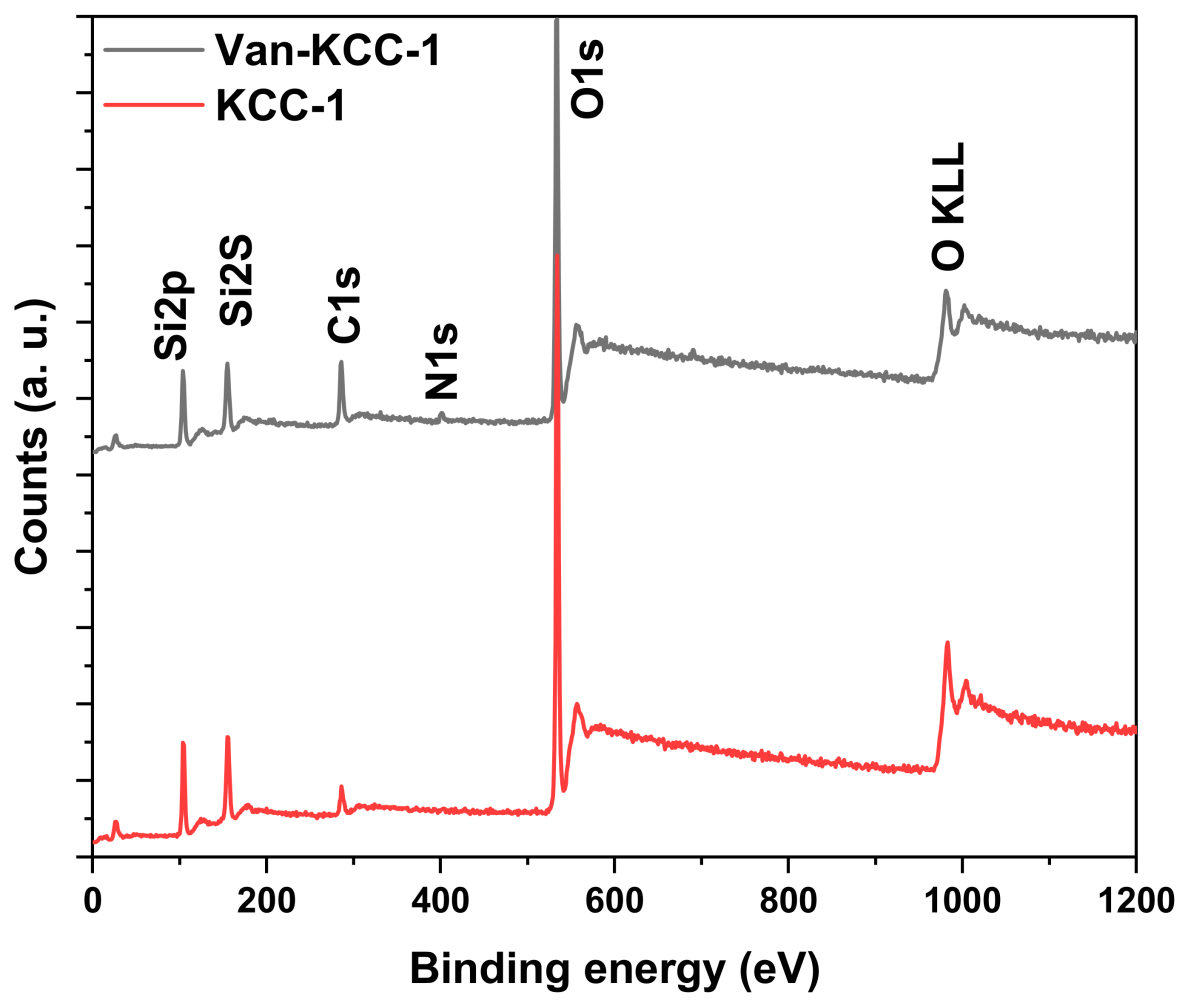
Comparative XPS survey spectra of KCC-1 and Van-KCC-1, showing the appearance of nitrogen and increased carbon intensity after Schiff-base functionalization.

X-ray photoelectron spectroscopy (XPS) was employed to verify the surface functionalization of KCC-1 with the o-vanillin Schiff-base ligand. As shown in the survey spectrum (Figure 4), the pristine silica framework is dominated by Si and O signals, whereas Van-KCC-1 exhibits a clear increase in carbon intensity together with the appearance of a nitrogen signal, confirming successful grafting of the organic Schiff-base moiety onto the silica surface. The predominance of Si 2p and O 1s peaks reflects the silica-rich composition of the support, while the relatively weak N 1s signal is expected because nitrogen originates only from the surface-bound organic layer and is therefore present at much lower concentration than the silica matrix.

**Figure 5:**
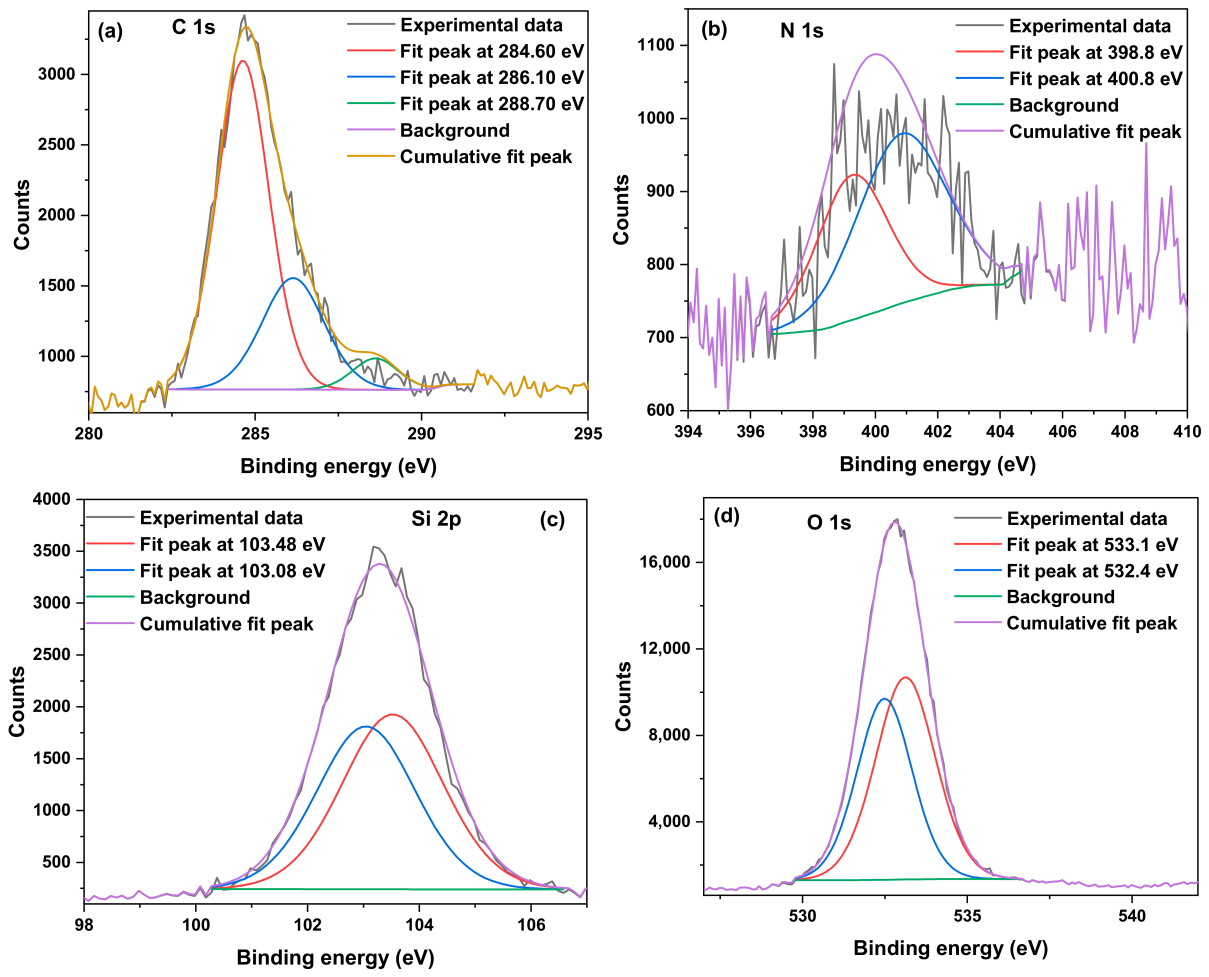
High-resolution XPS spectra of Van-KCC-1: C 1s (**a**), N 1s (**b**), Si 2p (**c**), and O 1s (**d**) regions. Each panel is labeled with the fitted component binding energies and their assignments, as detailed in the text.

The high-resolution spectra (Figure 5) further confirm the chemical structure of Van-KCC-1. Deconvolution of the C 1s region reveals three components centered at 284.6, 286.1, and 288.7 eV, assigned to C–C/C–H, C–O/C–N, and azomethine (C=N) carbon species, respectively (Figure 5a). The presence of the high-binding-energy C=N component provides direct evidence for Schiff-base formation following condensation between the amine-functionalized KCC-1 surface and o-vanillin. Consistently, the N 1s spectrum shows a dominant contribution at approximately 398.8 eV, characteristic of imine nitrogen (C=N), together with a weaker component near 400.8 eV that may arise from residual amine groups or partially protonated nitrogen species (Figure 5b). The observation of a strong imine-related N 1s signal corroborates the successful conversion of surface amine groups into Schiff-base functionalities.

The Si 2p spectrum displays the characteristic silica doublet centered at approximately 103.1 and 103.5 eV, consistent with Si(IV) species in the Si–O–Si network and indicating that the silica framework remains intact after chemical modification (Figure 5c). Likewise, the O 1s region is composed of contributions from silica oxygen atoms and oxygen-containing groups introduced by the ligand, including phenolic and methoxy functionalities (Figure 5d). The coexistence of imine carbon and nitrogen signals together with oxygen-containing organic species confirms the successful immobilization of the o-vanillin Schiff-base layer on the KCC-1 surface while preserving the structural integrity of the fibrous silica support.

**Figure 6:**
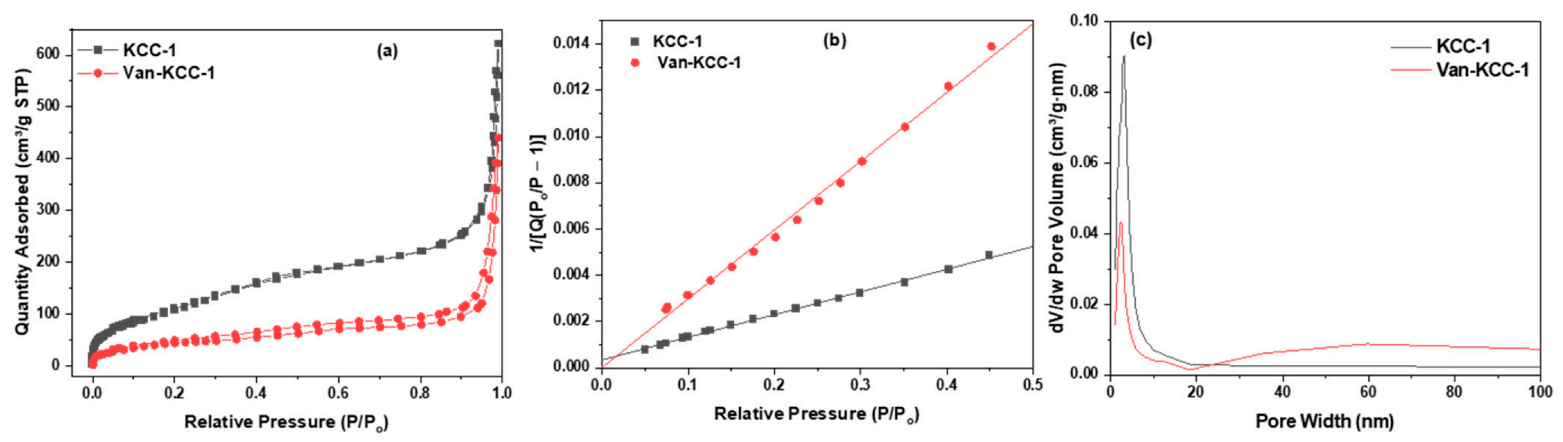
(**a**,**b**) N2 adsorption/desorption isotherms of pristine KCC-1 and Van-KCC-1 and (**c**) the BJH pore size distribution curves of the samples.

BET analysis was then used to quantify the effect of functionalization on surface area and porosity (Figure 6 and Table 1). For both KCC-1 and Van-KCC-1, the rapid increase in nitrogen adsorption at P/P_0_ > 0.95 is caused by capillary condensation in interparticle spaces rather than inside the intrinsic mesopores (Figure 6a). In order to prevent overestimation caused by external condensation, pore volumes were computed from the adsorption branch at P/P_0_ = 0.95. KCC-1 exhibited a BET surface area of 429 m^2^ g^−1^ Figure 6b), pore volume of 0.46 cm^3^ g^−1^, and BJH pore diameter of 3.15 nm (Figure 6c). After o-vanillin grafting, the corresponding values decreased to 260 m^2^ g^−1^, 0.19 cm^3^ g^−1^, and 2.30 nm. This decrease is expected for post-grafted silica because the organic layer occupies part of the accessible pore volume and increases wall thickness. Importantly, Van-KCC-1 still retained sufficient surface area and open mesoporosity for rapid analyte access. The reduction in surface area should therefore be interpreted together with the newly introduced binding chemistry: adsorption performance is governed by accessible fibrous channels and chemically suitable Schiff-base sites rather than by surface area alone.

The comparison between KCC-1, H2N-KCC-1, and Van-KCC-1 is interpreted as follows. Pristine KCC-1 provides the highest exposed silanol-rich surface but only weak, nonselective interaction with Ni(II) and Cd(II). H2N-KCC-1 introduces Lewis-basic amine sites and improves metal-ion affinity through electrostatic interaction and coordination. Van-KCC-1 further converts the amine anchor into an imine/phenolic ligand environment, producing a more selective hybrid organic-inorganic sorbent. The moderate reduction in BET surface area after functionalization is offset by the higher density and better chemical suitability of the active sites, which explains the improved analytical extraction performance. To isolate the contribution of the Schiff-base layer, Ni(II) and Cd(II) uptake by pristine KCC-1 and by H2N-KCC-1 was measured under identical conditions. Pristine KCC-1 gave low uptake through weak silanol interaction, H_2_N-KCC-1 gave intermediate uptake, and Van-KCC-1 gave the highest and most selective uptake. This control series shows that the imine and phenolic donor atoms, not the surface area, govern the adsorption of both metals.

Although static contact-angle measurements were not included in the original experimental plan, the interfacial behavior can be inferred from the surface chemistry. Calcined KCC-1 is strongly lyophilic/hydrophilic in water because of abundant silanol groups. Amination partially decreases surface polarity by introducing propyl chains, whereas Van-KCC-1 adds aromatic Schiff-base groups while retaining silanols and phenolic OH groups. Therefore, Van-KCC-1 is not lyophobic toward water; it remains sufficiently hydrophilic to disperse during DSPE, while the rough fibrous morphology increases effective solid–liquid contact. According to Wenzel-type wetting behavior, surface roughness amplifies the intrinsic wettability of a hydrophilic surface. The flower-like KCC-1 morphology is therefore expected to improve water penetration and analyte contact, supporting rapid extraction.

At the water-sorbent interface, Ni(II) and Cd(II) adsorption proceeds through coupled interfacial phenomena as follows: diffusion from the bulk solution to the external fibrous surface, electrostatic attraction or repulsion depending on pH, partial dehydration of the hydrated ions near the ligand sites, and surface complexation with imine/phenolic oxygen/nitrogen donor atoms. At low pH, protonation of the ligand sites suppresses adsorption. Near pH 5.0–6.0, partial deprotonation increases coordination ability without causing extensive metal-hydroxide precipitation. This balance explains the observed optimum pH and the efficient acid-driven desorption.

**Figure 7:**
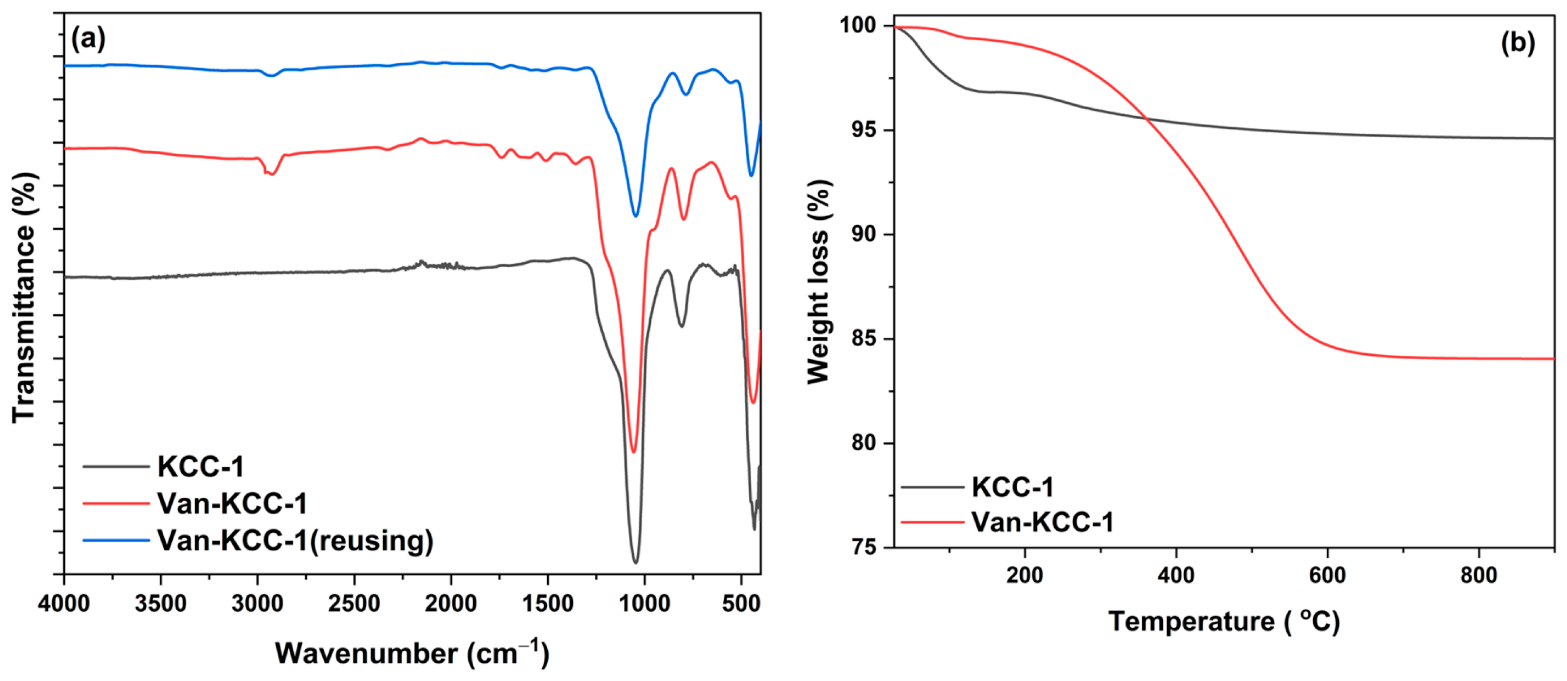
FT-IR spectra (**a**) and TGA curves (**b**) of calcined KCC-1 and Van-KCC-1.

FT-IR spectroscopy (Figure 7a) confirmed surface functionalization. KCC-1 showed broad absorption at 3200–3600 cm^−1^ from silanol groups and adsorbed water, along with Si-O-Si bands near 1060, 800, and 450 cm^−1^. After functionalization, Van-KCC-1 displayed additional organic bands, including aliphatic C-H stretching at 2926 and 2854 cm^−1^ and aromatic/imine-related bands in the 1600–1450 cm^−1^ region. The broad OH band may vary in intensity between KCC-1 and Van-KCC-1 because drying history, adsorbed atmospheric water, and organic grafting affect hydrogen bonding and water uptake. The presence of new organic bands, together with TGA and XPS evidence, confirms successful o-vanillin immobilization. The new bands were assigned as follows: aliphatic C-H stretching at 2926 and 2854 cm^−1^ from the APTES propyl chain, the C=N imine stretch near 1630 cm^−1^, aromatic C=C ring vibrations near 1600, 1580, and 1490 cm^−1^, and C-O stretching of the phenolic and methoxy groups near 1250 cm^−1^, which together confirm the grafted o-vanillin Schiff base. The silanol region of calcined KCC^−1^ appears weak and nearly flat rather than as a strong broad band, because calcination at 550 °C removes most physisorbed water and condenses surface silanols; the enlarged spectra in Figure 7a show this region more clearly.

TGA (Figure 7b) provided quantitative evidence of organic loading and stability. Both samples showed initial mass loss below 200 °C due to physisorbed water. Van-KCC-1 exhibited additional mass loss between 200 and 650 °C, assigned to decomposition of grafted propyl-imine/vanillin moieties. The absence of abrupt low-temperature decomposition indicates that the grafted layer is covalently bound and stable under the aqueous extraction conditions used in this study. The mass loss between 200 and 650 °C corresponds to an o-vanillin Schiff-base loading of approximately 0.8 to 1.0 mmol g^−1^, calculated from the organic weight loss and the ligand molar mass. This loading agrees with the reduction of BET surface area and pore volume after grafting, with the new C and N signals in XPS, and with the new organic bands in FT-IR, so the techniques give a single, internally consistent picture of covalent functionalization. The N 1s deconvolution also indicates that a small fraction of grafted APTES remained as free amine that did not condense with o-vanillin.

### 3.2. Adsorption Studies

The performance of Van-KCC-1 as a DSPE sorbent was evaluated through adsorption and analytical extraction experiments. The adsorption experiments clarify the metal-sorbent interaction, while the DSPE experiments define the final sample-preparation conditions used before ICP-OES measurement. This distinction is important because high adsorption capacity alone is insufficient for DSPE; quantitative desorption, matrix tolerance, low leaching, and reproducible recovery are also required.

#### 3.2.1. Effect of pH

**Figure 8:**
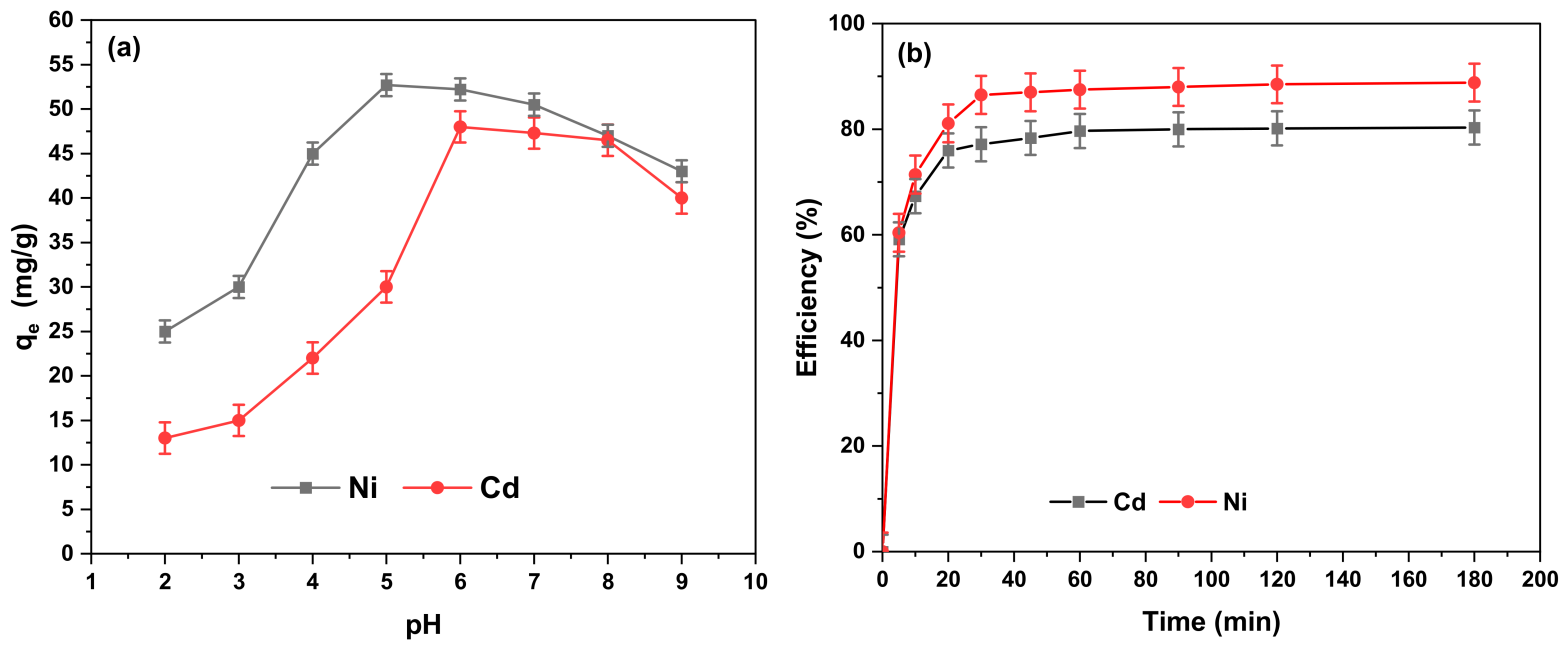
Effect of pH (**a**) and contact time (**b**) on Ni(II) and Cd(II) adsorption onto Van-KCC-1. Data are presented as mean ± SD (*n* = 3). Initial concentration = 60 mg L^−1^, contact time = 90 min for pH study, adsorbent dosage = 0.1 g, temperature = 25.5 °C.

The pH of solution controls metal-ion speciation, protonation of the Schiff-base sites, and possible metal-hydroxide precipitation. Adsorption increased from pH 2.0 to 5.0–6.0 and decreased at higher pH (Figure 8a). At acidic pH, protonated imine and phenolic sites repel cationic analytes and compete with metal ions for binding. Near pH 5.0–6.0, deprotonated donor atoms promote coordination with Ni(II) and Cd(II). At higher pH, the formation of amino complexes of Ni(II) and Cd(II) likely interferes with their interaction with the Schiff-base grafted on the porous material, so removal may no longer represent true sorption. A common DSPE pH of 5.0 was therefore selected to provide high recoveries for both analytes and avoid precipitation artifacts.

#### 3.2.2. Adsorption Kinetics

The dendritic fibrous morphology of KCC-1, combined with the high accessibility of Schiff-base sites, produced rapid adsorption kinetics. Equilibrium was reached within 60–90 min (Figure 8b), with about 85 to 90% of the equilibrium capacity attained during the first 30 min. This early uptake is analytically important because DSPE methods are commonly constrained by short vortexing or shaking times. The result indicates that the external fibrous surface contributes substantially to the initial uptake and that the functionalized layer remains accessible after grafting. Such fast uptake is advantageous for vortex-assisted DSPE, where short contact times directly improve sample throughput without sacrificing recovery [15,16,17,18,19].

**Figure 9:**
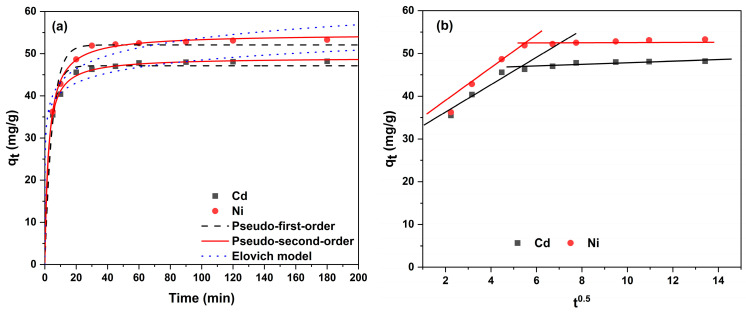
(**a**) Adsorption kinetic profiles for Ni(II) and Cd(II) on Van-KCC-1 fitted with pseudo-first-order [27], pseudo-second-order [28], and Elovich [29]. (**b**) Data fitting with the intraparticle diffusion [30] models and experimental data. Data are presented as mean ± SD (*n* = 3). Initial concentration = 60 mg L^−1^, adsorbent dosage = 0.1 g, temperature = 25.5 °C.

Kinetic data were fitted to the pseudo-first-order (PFO) [27], pseudo-second-order (PSO) [28], Elovich [29], and intraparticle diffusion (IPD) [30] models (Figure 9 and Table 2). These models were selected because they test different kinetic assumptions as follows: PFO describes uptake controlled mainly by the number of unoccupied sites, PSO is commonly used to describe chemisorption-controlled processes, the Elovich model reflects energetically heterogeneous surfaces, and IPD evaluates diffusion contribution within pores or particle domains. The PSO model provided the best correlation (R^2^ ≥ 0.998) and the lowest RMSE values, and the calculated equilibrium capacities (49.08 mg g^−1^ for Ni(II) and 54.67 mg g^−1^ for Cd(II)) closely matched the experimental capacities (Figure 9a). This agreement supports a surface-complexation-controlled process involving the imine/phenolic groups and metal centers [23,31]. Multi-linear IPD plots (Figure 9b) that did not pass through the origin confirmed that intraparticle diffusion contributes to uptake but is not the sole rate-limiting step. The initial steep segment reflects rapid boundary-layer diffusion onto abundant external fibrous sites, while the later segment reflects slower diffusion into mesopores. These mixed-rate characteristics validate the suitability of Van-KCC-1 for kinetically demanding DSPE workflows.

#### 3.2.3. Adsorption Isotherms

**Figure 10:**
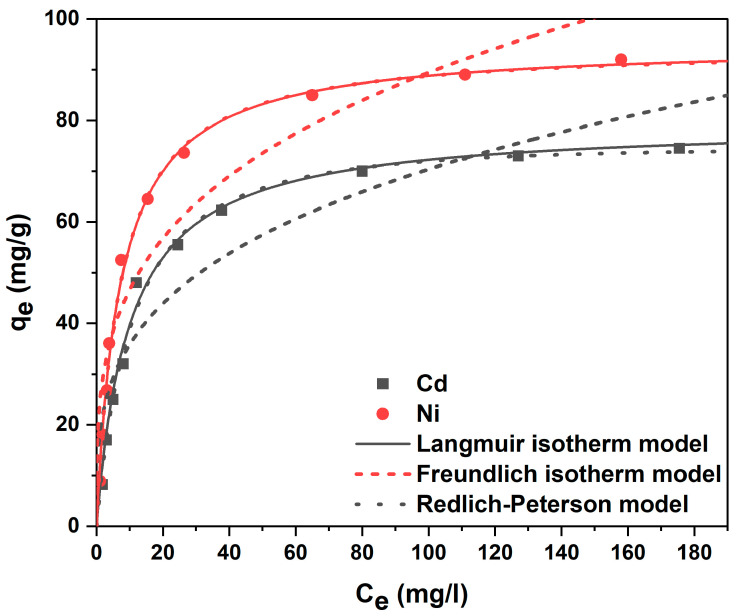
Adsorption isotherms for Ni(II) and Cd(II) on Van-KCC-1 fitted with Langmuir [33], Freundlich [34], and Redlich–Peterson [35] models. Data are presented as mean ± SD (*n* = 3). Contact time = 90 min, adsorbent dosage = 0.1 g, temperature = 25.5 °C. Concentration units are mg L^−1^.

Equilibrium data were modeled using the Langmuir [32], Freundlich [33], and Redlich–Peterson (R-P) [34] isotherms (Table 3) by nonlinear regression (Figure 10). The three models were chosen to distinguish between ideal monolayer adsorption, heterogeneous multilayer-type adsorption, and hybrid behavior. The Langmuir model gave the highest correlation coefficients (R^2^ ≥ 0.991) and the lowest RMSE values, indicating dominant monolayer coverage on a finite number of accessible binding sites. Maximum monolayer capacities (q_max_) were 79.45 mg g^−1^ for Cd(II) and 95.19 mg g^−1^ for Ni(II) at 298 K and the respective optimum pH values. On pristine calcined KCC-1, same batch adsorption studies were carried out. The experimental q_max_ values were more than seven times lower than Van-KCC-1, at 8.4 mg g^−1^ (Cd) and 11.2 mg g^−1^ (Ni). The dramatic increase demonstrates that the imine/phenolic donor set, rather than silanols, dominates uptake. The Redlich–Peterson exponent beta approached unity (1.00–1.04), which further supports Langmuir-type behavior rather than strongly heterogeneous adsorption. In contrast, the Freundlich model showed poorer fitting, as reflected by higher RMSE values. The attained capacities are superior or comparable to those reported for other Schiff-base-functionalized mesoporous silicas [35,36,37,38,39], while the moderate binding affinity remains compatible with rapid acid desorption required for DSPE operation.

Together, the pH, kinetic, and isotherm data confirm that Van-KCC-1 functions as a high-capacity and kinetically favorable DSPE sorbent. The optimum pH provides sufficient ligand deprotonation without extensive hydroxide precipitation. The PSO fit supports surface-complexation-controlled uptake, whereas the Langmuir fit indicates that accessible Schiff-base sites dominate the equilibrium behavior. This mechanistic consistency is important because it links the material design to the analytical outcome: the sorbent provides sufficient affinity for trace-level extraction, while acid desorption remains efficient because the coordination sites are moderate rather than irreversible.

### 3.3. DSPE Application

The synthesized Van-KCC-1 was applied as a DSPE sorbent for the extraction of Ni(II) and Cd(II) from aqueous samples, prior to their determination by ICP-OES. The performance of the method was evaluated in terms of calibration characteristics and applicability to real water samples.

#### 3.3.1. Calibration Characteristics of the DSPE-ICP-OES Method

In this method, DSPE was used as a sample-preparation step for preconcentration and partial matrix elimination before ICP-OES determination. Calibration curves were prepared after the complete extraction procedure rather than by direct instrumental calibration. This approach was intentionally selected because it incorporates extraction recovery, desorption efficiency, phase separation, and matrix transfer into the calibration response. Linear ranges were 0.035–50 µg L^−1^ for Ni(II) and 0.058–50 µgL^−1^ for Cd(II), with correlation coefficients of 0.997 and 0.994, respectively (Table 4). LODs were 0.011 µgL^−1^ for Ni(II) and 0.019 µg L^−1^ for Cd(II), and LOQs were 0.035 and 0.058 µg L^−1^, respectively. The average RSD values were 3.10% and 3.60%, demonstrating good repeatability under the optimized DSPE conditions. The actual preconcentration factors (9.60 for Ni(II) and 9.30 for Cd(II)) were close to the theoretical factor of 10, confirming that the selected elution volume and desorption conditions were appropriate.

#### 3.3.2. Application to Real Water Samples

We evaluated the developed DSPE method, coupled to ICP-OES, using NIST CRM 1643d and real water samples. The certified reference material was used to test the complete analytical workflow rather than only the instrumental measurement. The determined concentrations of Ni(II) and Cd(II) were in good agreement with the certified values (Table 5), confirming method accuracy after extraction, desorption, and ICP-OES measurement. Matrix-spike recovery experiments were then performed by spiking mineral water, tap water, and synthetic wastewater samples with known analyte concentrations before extraction (Table 6).

The obtained recoveries ranged from 92.00% to 97.00% for real water matrices, with RSD values below 4.78% (Table 6). These values confirm that dissolved salts and simulated wastewater constituents did not prevent adsorption or desorption under the optimized conditions. The consistent recoveries across sample types agree with the interference study (Table 7) and show that Van-KCC-1 can be used for trace-level analysis in realistic water matrices.

These findings confirm that Van-KCC-1 is effective as a DSPE sorbent for sample preparation, enabling accurate and reproducible determination of trace metal ions in environmental water samples.

### 3.4. Reusability and Interference Studies

**Figure 11:**
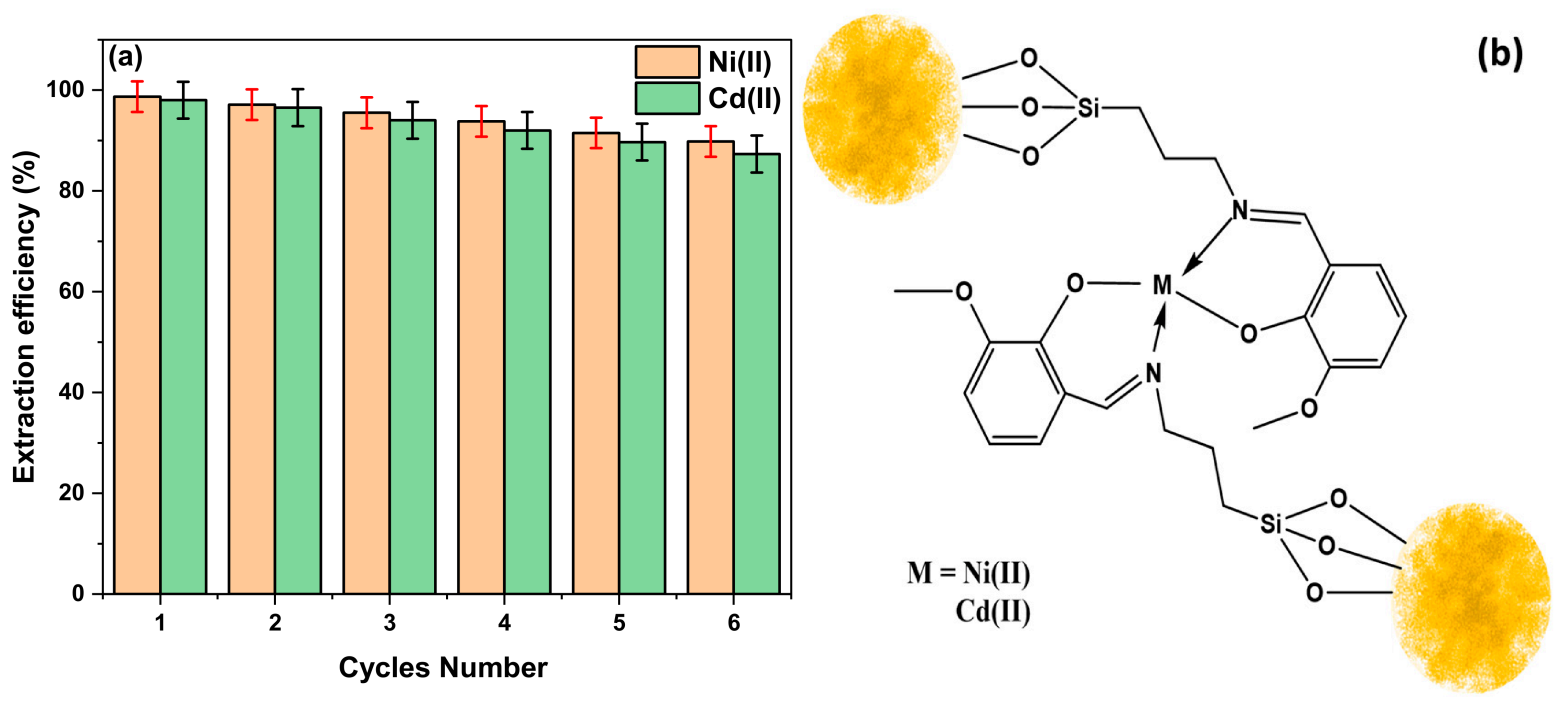
(**a**) Reusability of Van-KCC-1 adsorption–desorption cycles. (**b**) Proposed interaction between the o-vanillin Schiff-base sites of Van-KCC-1 and Ni(II)/Cd(II).

Van-KCC-1 retained over 87% extraction efficiency after six consecutive adsorption–desorption cycles using 0.1 M HNO3 regeneration for 5 min, demonstrating practical reusability (Figure 11a). The retained efficiencies after the sixth cycle were 89.80% for Ni(II) and 87.30% for Cd(II). This behavior indicates that the Schiff-base layer is stable enough for repeated acidic elution under the selected DSPE conditions. The effect of common coexisting ions (NH^4+^, Na^+^, K^+^, Ca^2+^, Mg^2+^, Cr^3+^, Fe^3+^, CO_3_^2−^, SO_4_^2−^, and Cl^−^) was evaluated at high ion-to-analyte ratios (Table 7). The results show that common matrix ions caused limited signal change, supporting the selectivity of Van-KCC-1 for the target ions. From an analytical perspective, this outcome is essential because natural waters contain major ions at concentrations far higher than trace Ni(II) and Cd(II). The extraction efficiency was recorded after each of the six adsorption–desorption cycles and is shown in the new reusability figure; the efficiency decreased only gradually, reaching 89.80% for Ni(II) and 87.30% for Cd(II) at the sixth cycle. Simple aliphatic imines hydrolyse readily in acid, but the ortho-hydroxy aryl imine formed from o-vanillin is a salicylaldimine-type ligand that is stabilized by intramolecular O-H to N hydrogen bonding and by chelate resonance, which raises its resistance to hydrolysis. The acid contact during elution is short (0.1 M HNO3, 5 min) and is followed by washing, which further limits hydrolytic loss. After adsorption–desorption cycles, the structural stability of the reused Van-KCC-1 was evaluated by comparing its FT-IR spectra with that of the new catalyst (Figure 7a). The intensity of the Schiff-base-related bands decreased slightly as follows: C=N stretching (1620–1640 cm^−1^), aromatic C=C (1500–1600 cm^−1^), phenolic C–O (1250–1270 cm^−1^), and aliphatic C–H (2920–2850 cm^−1^). In addition to slight surface deactivation or incomplete desorption during regeneration, interactions with adsorbed molecules are responsible for this reduction. The fibrous KCC-1 structure was confirmed by the fact that the silica framework bands—Si–O–Si stretching (~1060 cm^−1^), symmetric Si–O (800 cm^−1^), and Si–O bending (450 cm^−1^)—remained virtually unaltered. The absence of significant peak changes or new absorption bands further implies that the Schiff-base-functionalized silica preserved its chemical integrity, demonstrating the high stability and reusability of Van-KCC-1. The imine (Schiff base, –C=N–) linkage is unstable under strongly acidic conditions, as supported by both mechanistic understanding and extensive experimental evidence. Previous studies have shown that metal cations readily desorb from Schiff base-functionalized silica nanoparticles in strong acids such as nitric, sulfuric, and hydrochloric acid. Building on this, we investigated the desorption process using nitric acid at concentrations of 0.05 M, 0.1 M, and 1 M at room temperature [8,19].

### 3.5. Selectivity, Leaching, and Interfacial Stability

The presence of Ca(II) and Mg(II) is particularly relevant because natural waters normally contain these ions at concentrations much higher than trace Ni(II) and Cd(II). Their limited effect on recovery indicates that Van-KCC-1 does not behave as a purely nonspecific cation exchanger. Instead, the imine/phenolic ligand environment favors transition-metal coordination over alkaline-earth binding. Cr(III) and Fe(III) produced slightly stronger interference because they can also coordinate to oxygen/nitrogen donor atoms; however, recoveries remained acceptable under the tested ratios. This selectivity pattern supports the proposed role of Schiff-base donor atoms in the extraction mechanism. A schematic of the proposed interaction is shown in Figure 11b, in which the imine nitrogen and the phenolic oxygen of each o-vanillin unit coordinate Ni(II) or Cd(II) in a bidentate manner. The effective acid elution and reversible binding are due to the protonation of the donor atoms in dilute nitric acid. This protonation weakens the metal–ligand interaction and releases the metal ions during the desorption step. 

Possible leaching from KCC-1, H2N-KCC-1, and Van-KCC-1 was considered because ligand loss can compromise reusability, blank levels, and analytical accuracy. The following several observations indicate that leaching was not significant under the optimized conditions: (i) Van-KCC-1 retained high extraction efficiency after six acid-regeneration cycles; (ii) procedural blanks remained low; (iii) recoveries in NIST CRM 1643d and real samples were accurate; and (iv) TGA and XPS confirmed covalent organic grafting rather than simple physical adsorption. These findings support the interfacial stability of the sorbent during repeated adsorption and acid-desorption cycles. The post-cycle FT-IR results described above, together with the low procedural blanks, confirm the chemical integrity of the Schiff-base layer after repeated reuse (Figure 7a).

### 3.6. Comparison with Other Adsorbents and with AAS-Based Methods

Table 8 compares the proposed Van-KCC-1 sorbent with representative modified adsorbents reported for aquatic pollutants and trace-metal extraction. The comparison was included to evaluate the method by analytical criteria rather than by adsorption capacity alone. Van-KCC-1 offers a balanced performance profile as follows: low detection limits, short extraction/desorption time, good reusability, and acceptable matrix tolerance. Compared with conventional AAS-based workflows, ICP-OES provides simultaneous multi-element capability, a wider linear dynamic range, and higher sample throughput. Flame AAS is often less sensitive for trace Cd(II) and Ni(II), whereas graphite furnace AAS can achieve low detection limits but generally measures one element at a time and requires longer furnace programs. The DSPE-ICP-OES combination is therefore advantageous when rapid routine monitoring of multiple trace metals is required. On an item-by-item basis, Van-KCC-1 compares as follows. Its capacities (79.45 mg g-1 for Cd(II) and 95.19 mg g-1 for Ni(II)) are comparable to those of organically modified Schiff-base silica aerogel. Then, (49.9 mg g-1 for Ni(II)) and tetradentate Schiff-base functionalized ion-imprinted silica (31.4 mg g-1 for Cd(II)) [41,42]. Soltani and colleagues [43] used ultrasonic-assisted in situ ring-opening polymerization in solvent-free conditions to produce a carboxylic acid-functionalized KCC-1/polyamide 6 nanocomposite (COOH-KCC-1/PA6 NC). The substance had a high potential for heavy metal adsorption due to its abundance of carboxylic, secondary amine, and ketone groups. It reached a maximum adsorption capacity (q_max_) of 109.2 mg/g for Cd(II) under ideal conditions (50 mg adsorbent, pH 7.0, 80 mg/L starting Cd(II) concentration, 298 K, and 240 min contact time). Its DSPE-ICP-OES detection limits (0.011 and 0.019 microgram L^−1^) are comparable to Schiff-base silica gel (39.5 ng L^−1^ for Ni and 28.2 ng L^−1^ for Cd) and lower than typical flame-AAS workflows. Its extraction and desorption time (about 20 min extraction and 5 min elution) is shorter than packed-bed SPE, its recoveries (92 to 99%) match the best silica sorbents, and its reusability (more than 87% after six cycles) exceeds strongly chelating sorbents that need longer or stronger elution. These figures, taken from the references in Table 8, support the comparison on every indicator rather than on capacity alone.

## 4. Conclusions

A novel Schiff-base-functionalized dendritic fibrous silica nanomaterial (Van-KCC-1) was successfully synthesized through sequential amination of KCC-1 with APTES followed by grafting of o-vanillin. The characterization by XRD, BET, FE-SEM/TEM, FT-IR, TGA, and XPS confirmed successful surface functionalization while preserving the characteristic fibrous architecture of KCC-1. Although organic modification reduced the specific surface area, it generated a high density of accessible coordination sites without compromising the open radial channel structure responsible for rapid mass transfer. The resulting nanomaterial exhibited favorable adsorption behavior toward Cd(II) and Ni(II), following pseudo-second-order kinetics and Langmuir-type adsorption with maximum capacities of 79.45 and 95.19 mg g^−1^, respectively. When employed as a dispersive solid-phase extraction sorbent coupled with ICP-OES, Van-KCC-1 enabled sensitive and accurate determination of both analytes, providing low detection limits, wide linear ranges, satisfactory precision, and recoveries of 92–98% in certified reference material and diverse water matrices. The sorbent also displayed good selectivity in the presence of common coexisting ions and retained more than 87% of its initial extraction efficiency after six adsorption–desorption cycles, confirming its stability and reusability. These properties arise from the synergistic combination of the dendritic fibrous KCC-1 framework, which facilitates analyte transport and accessibility, and the o-vanillin-derived Schiff-base functionality, which provides selective yet reversible coordination sites for metal-ion binding. The integration of o-vanillin-derived imine, phenolic, and methoxy donor groups within the KCC-1 nanostructure yielded a sorbent that combines efficient extraction, quantitative desorption, and operational stability. This balance between adsorption strength and reversibility is particularly advantageous for analytical sample-preparation workflows, where both quantitative recovery and sorbent regeneration are essential. The findings highlight the potential of fibrous mesoporous silica nanomaterials as versatile platforms for the development of reusable nanochelators for trace-metal monitoring and related separation applications.

## Figures and Tables

**Table 1:** Textural properties of pristine KCC-1 and Van-KCC-1 according to the BET and BJH equations represent the surface area (m^2^g^−1^), pore volume (cm^3^ g^−1^), and pore diameter (nm), respectively).

Sample	BET Surface Area (m^2^/g)	Pore Volume (cm^3^/g)	Most Probable Pore Diameter (nm)	Particle Size (nm)
KCC-1	429	0.46	3.15	330
Van-KCC-1	260	0.19	2.3	370

**Table 2:** Kinetic adsorption parameters and RMSE for Cd(II) and Ni(II) adsorption onto Van-KCC-1 at 298 K.

Models	Parameters	Cd(II)	Ni(II)
Pseudo-first-order [27]	qe,cal (mg g^−1^)	47.13	52.07
	k1 (min^−1^)	0.25	0.209
	R	0.993	0.989
	RMSE	1.46	1.73
Pseudo-second-order [28]	qe,cal (mg g^−1^))	49.08	54.67
	k2 (g mg^−1^ min^−1^)	0.0105	0.0072
	R	0.999	0.998
	RMSE	0.77	0.56
Elovich [29]	α (mg g^−1^ min^−1^)	5.74 × 10^4^	6.06 × 10^3^
	β (g mg^−1^)	0.29	0.22
	R	0.984	0.979
	RMSE	0.43	0.68
Intraparticle diffusion [30]	kid (mg g^−1^ min^−1/2^)	3.41	4.26
	C (mg g^−1^)	28.88	28.33
	R	0.93	0.97
	RMSE	1.62	1.55

**Table 3:** Adsorption isotherm parameters and RMSE for Cd(II) and Ni(II) adsorption onto Van-KCC-1 at 298K.

Models	Parameters	Cd(II)	Ni(II)
Langmuir [32]	q_max_ (mg g^−1^)	79.45	95.19
	K_L_ (L mg^−1^)	0.1	0.14
	R	0.991	0.994
	RMSE	2.61	2.47
Freundlich [33]	K_F_(mgg^−1^)/(mgL^−1^)^(1/n)^	18.22	24.35
	n (dimensionless)	3.41	3.56
	R	0.88	0.89
	RMSE	8.92	10.88
Redlich–Peterson [34]	KR-P (L g^−1^)	6.97	13.08
	αR-P((mgL^−1^)β)	0.07	0.135
	β (dimensionless)	1.03	1.04
	R	0.992	0.994
	RMSE	2.53	2.64

**Table 4:** Analytical characteristics of the proposed DSPE-ICP-OES method.

Calibration Parameter	Ni(II)	Cd(II)
Linearity, μg/L	0.035–50	0.058–50
Correlation coefficient (R^2^)	0.997	0.994
Limit of detection (LOD, μg/L)	0.011	0.019
Limit of quantification (LOQ, μg/L)	0.035	0.058
Average relative standard deviation (RSD %)	3.10	3.60
Average recovery (%)	97.30	95.40
Preconcentration factor (PF) (theoretical)	10	10
Preconcentration factor (PF) (actual)	9.60	9.30

**Table 5:** Determination of Cd(II) and Ni(II) in NIST CRM 1643d.

Metal Ions	Certified (μg L^−1^)	Found (μg L^−1^)	Recovery (%)
Cd(II)	6.47 ± 0.37	6.25 ± 0.3	96.61
Ni(II)	58.1 ± 0.27	57.42 ± 0.91	98.83

**Table 6:** Recoveries of Cd(II) and Ni(II) from real water samples (*n* = 3).

Sample	Added (μg/L)	Found (Microgram L^−1^)		RSD (%)		Recovery (%)	
		Ni(II)	Cd(II)	Ni(II)	Cd(II)	Ni(II)	Cd(II)
Mineral water	-	0.00	0.00				
	5	4.85 ± 0.15	4.75 ± 0.16	3.10	3.36	97.00	95.00
	10	9.50 ± 0.23	9.40 ± 0.26	2.40	2.77	95.00	94.00
Tap water	-	0.20	0.15				
	5	4.75 ± 0.16	4.67 ± 0.20	3.36	4.28	95.00	93.40
	10	9.35 ± 0.40	9.28 ± 0.44	4.27	4.74	93.50	92.80
Synthetic waste water *	-	0.00	0.00				
	5	4.65 ± 0.19	4.62 ± 0.20	4.08	4.30	93.00	92.40
	10	9.26 ± 0.40	9.20 ± 0.44	4.32	4.78	92.60	92.00

* Composition based on Shrestha et al. 2023 [40].

**Table 7:** Effect of coexisting ions on the recoveries of Ni(II) and Cd(II) (50 microgram L^−1^, *n* = 3).

Interference	Interference to Metal Ion Ratio	Recovery (%)	
		Ni(II)	Cd(II)
NH4^+^, Na^+^, K^+^	1000	97.3	95.7
Ca^2+^	50	97.0	96.78
Mg^2+^	75	95.2	93.08
Cr^3+^	5	94.2	91.5
Fe^3+^	3	93.1	90.6
CO_3_^2−^, SO_4_^2−^, Cl^−^	1000	97.8	95.91

**Table 8:** Comparative summary of the proposed Van-KCC-1 sorbent and selected mesoporous or functionalized adsorbents reported for aquatic pollutant removal and trace-metal extraction.

Sorbent/Material	Target Analyte(s)	Main Advantage	Main Limitation	q_max_ (mg/g)	Reference
MCM-48 nanoporous silica	Aniline/nitroanilines	3D ordered mesopores; useful aquatic pollutant adsorbent	Organic-pollutant focus; not trace-metal DSPE	94	[14]
SBA-15-based mesoporous adsorbents	Cd(II) Ni(II)	High area and tunable cylindrical mesopores	Diffusion can be affected by channel blockage after grafting	7.9 16.7	[20,21]
Bio-originated cyclam-SBA-15	Ni(II)	High Ni(II) adsorption capacity and green silica source	Strong chelation may require stronger/longer elution for analytical reuse	243.36	[44]
Amine-functionalized KCC-1	Cd(II)	Open fibrous morphology and rapid microextraction	Amine sites provide lower ligand specificity than Schiff-base sites	LOD = 0.02 µg/L	[22]
Van-KCC-1	Ni(II) Cd(II)	Open fibrous KCC-1 plus reversible imine/phenolic binding; low LODs by DSPE-ICP-OES	Contact-angle and direct leachate TOC tests can be added in future work	95.19 79.45	This study

## Data Availability

The original contributions presented in this study are included in the article. Further inquiries can be directed to the corresponding author.
